# Supplementary material for: Automated Extraction of Mortality Information From Publicly Available Sources Using Large Language Models: Development and Evaluation Study
Source: J Med Internet Res. 2025 Aug 18;27:e71113. doi: 10.2196/71113 (PMC12359966; doi:10.2196/71113)
Supplement: Multimedia Appendix 1 [file jmir-v27-e71113-s001.docx]

**1.1 Metrics definition and measurement**

**Recall (Sensitivity)**
Sensitivity measures the ability of the model to correctly identify true positive cases. It is calculated as:
  *Sensitivity = True Positives (TP) / (TP + False Negatives (FN))*
In this context, it reflects the proportion of relevant mortality-related details that the model successfully extracts.

**Precision (Positive Predictive Value, PPV)**
Precision evaluates the accuracy of the positive predictions made by the model. It is given by:
  Precision = TP / (TP + False Positives (FP))
This metric indicates the proportion of items identified by the model as positive that are truly positive.

**Accuracy**
Accuracy represents the overall correctness of the model by calculating the proportion of total predictions (both positive and negative) that are correct. It is defined as:
  *Accuracy = (TP + True Negatives (TN)) / Total Number of Cases*

**F1-Score**
The F1-score is the harmonic mean of precision and recall, offering a single measure of a model’s performance when balancing precision and sensitivity. It is calculated using:
*F1-Score = 2 × (Precision × Recall) / (Precision + Recall)*

**Information Density**
Information density assesses whether an online post contains sufficient details for reliable patient linkage. In our study, it is defined as the percentage of posts in which a key identifier (e.g., an annotated decedent name) is present. For example, if 90 out of 100 posts contain an annotated name, the information density is calculated as:
  Information Density (%) = (Number of Posts with Annotated Names / Total Number of Posts) × 100

**1.2 Confusion matrices for the finetuned transformer models on the named entity recognition tasks (Decedent Name, Date of Death, and Date of Birth)**


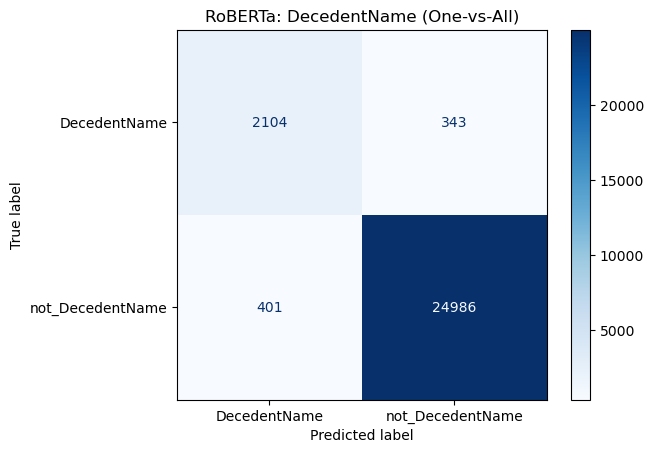

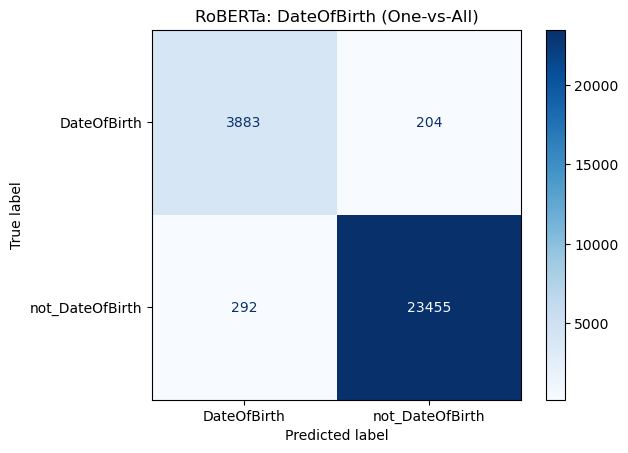


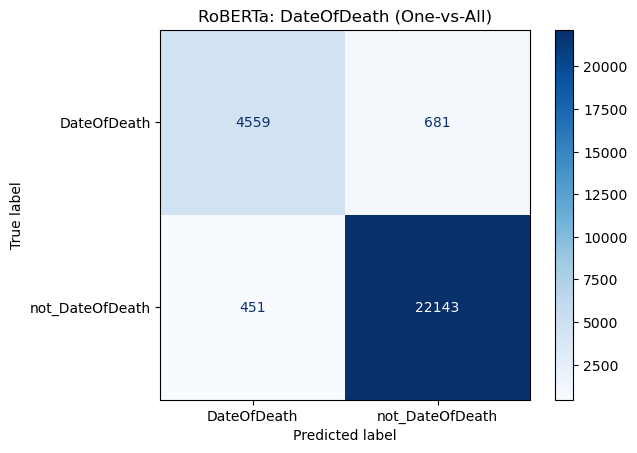

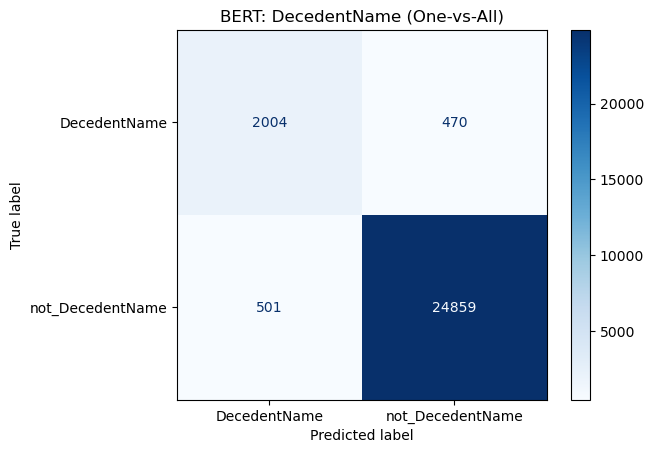


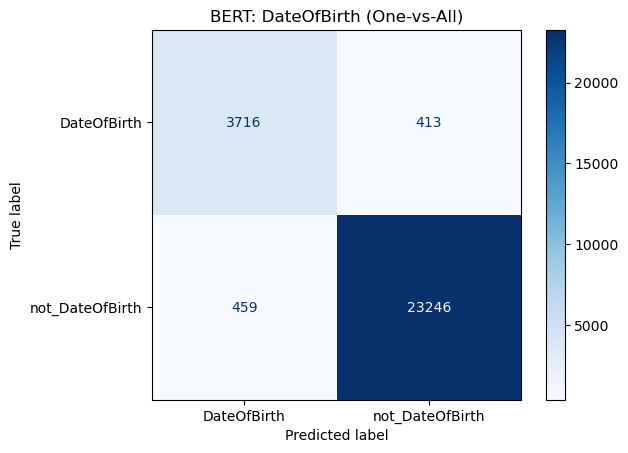

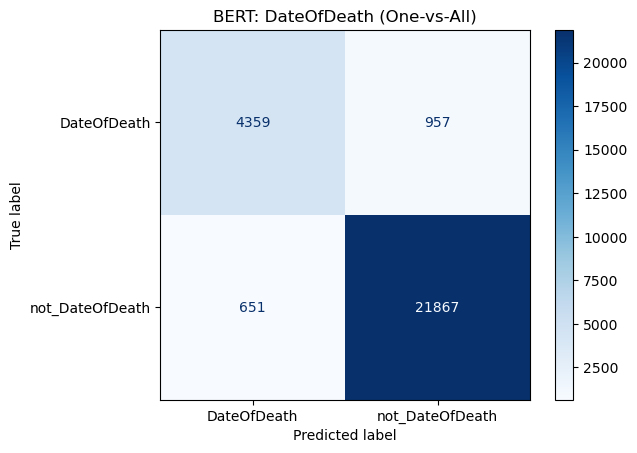


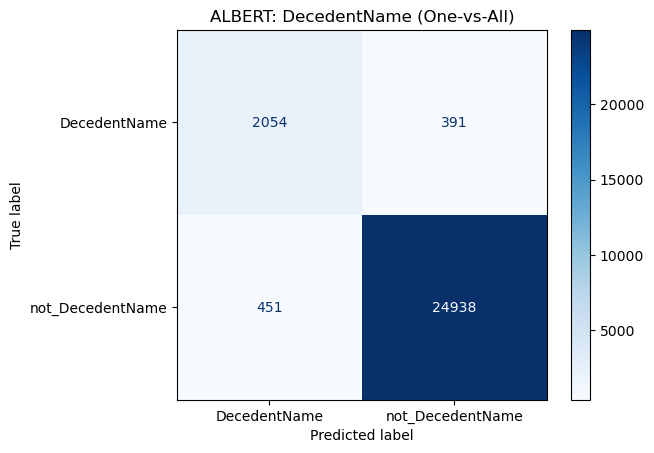

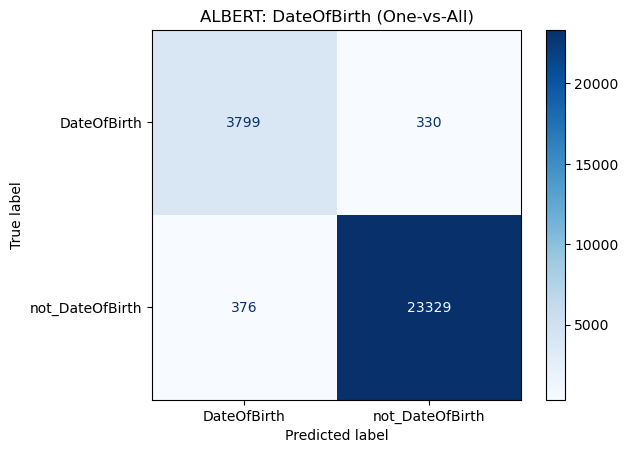


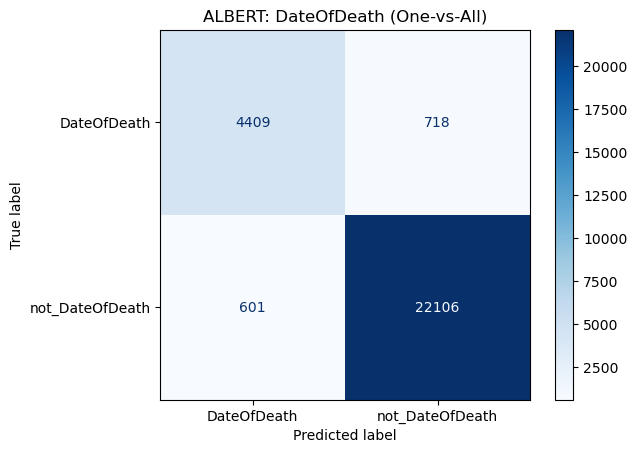

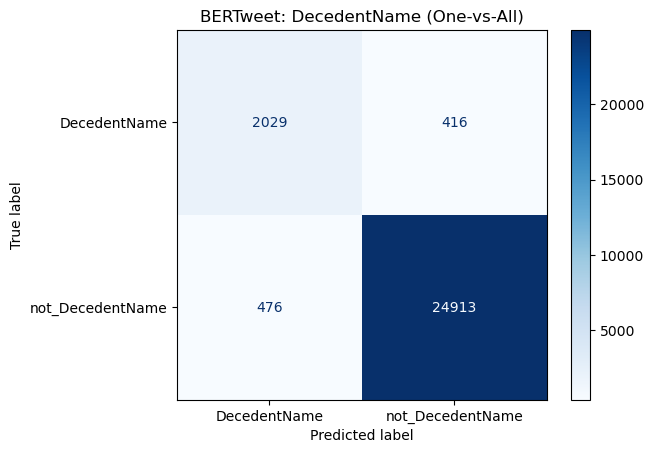


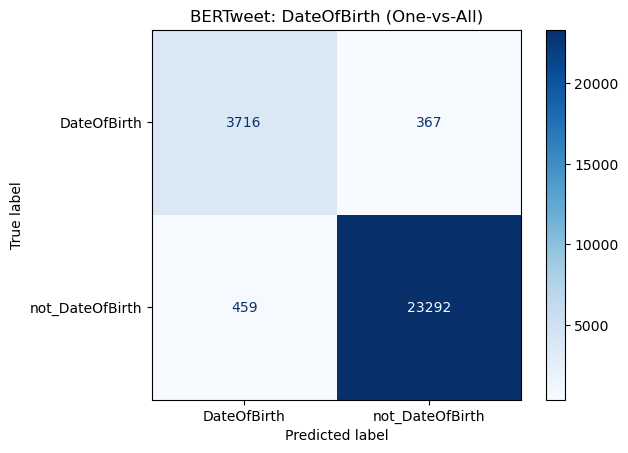

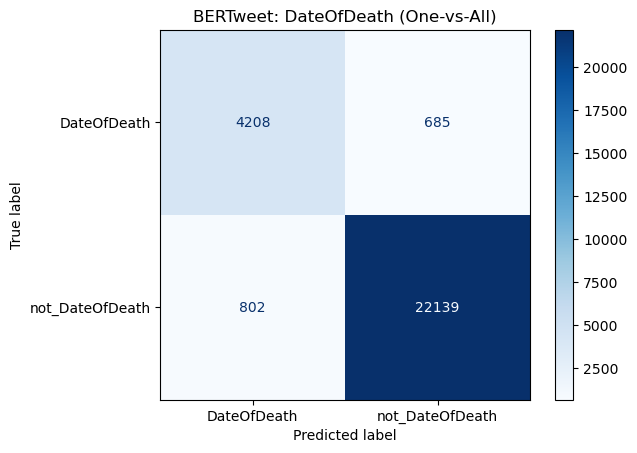


​

**3.1 LLM-Prompt example**

You are provided with examples that map public posts to their respective causes of death. Use these examples as guidance. Ignore any informal language, slang, or extraneous noise, and focus on the essential medical details.

Definitions:

- Primary Cause of Death: The condition that initiated the chain of events leading directly to death.

- Secondary Cause of Death: A contributing factor that did not initiate the fatal sequence. If not mentioned, label it as "Not Mentioned".

Example 1:

Post: "We are heartbroken—our dad, Jim, fought lung cancer for years but eventually lost his battle due to complications."

Primary Cause: Lung Cancer

Secondary Cause: Not Mentioned

Example 2:

Post: "Our family is heartbroken. Our dear mother had battled heart disease for years, and a sudden heart attack took her from us unexpectedly."

Primary Cause: Heart Disease

Secondary Cause: Not Mentioned

Example 3:

Post: "OMG, we're devastated. Our Uncle Mike was a total warrior – he had been battling stage 4 liver cancer for months and even went through grueling surgery. But then, out of nowhere, he got hit with a brutal bout of pneumonia right after his operation. The pneumonia hit him hard, and despite all the medical care, his condition spiraled out of control, ultimately taking his life. Our hearts are shattered."

Primary Cause: Pneumonia

Secondary Cause: Liver Cancer

.

.

.

.

Example(n)

**1.4 Latest Refined Keywords Indicating a Deceased Person**

1. death
2. expired
3. deceased
4. passed away
5. no longer with us
6. rest in peace
7. RIP
8. tragic loss
9. sudden death
10. untimely death
11. fatality
12. perished
13. Honoring memory
14. last breath
15. in memoriam
16. obituary
17. mourning
18. bereavement
19. condolence
20. in loving memory
21. remembrance
22. tribute
23. grief
24. gone too soon
25. forever in our hearts
26. never forgotten
27. life well lived
28. celebrating life
29. missed but not forgotten
30. angel now
31. heaven gained an angel
32. remembering you
33. legacy lives on
34. Memories live on
35. Always in our hearts
36. memorial moment
37. tribute today
38. honoring memory
39. digital memorial
40. virtual candle
41. online vigil
42. memory wall
43. grief journey
44. support in sorrow
45. Gone but never forgotten
46. heartbroken but strong
47. community mourns
48. collective grief
49. rest in power
50. funeral announcement
51. celebration of life ceremony
52. Forever missed
