# Supplementary figures and images for: Automated Extraction of Mortality Information From Publicly Available Sources Using Large Language Models: Development and Evaluation Study
Source: J Med Internet Res. 2025 Aug 18;27:e71113. doi: 10.2196/71113 (PMC12359966; doi:10.2196/71113)

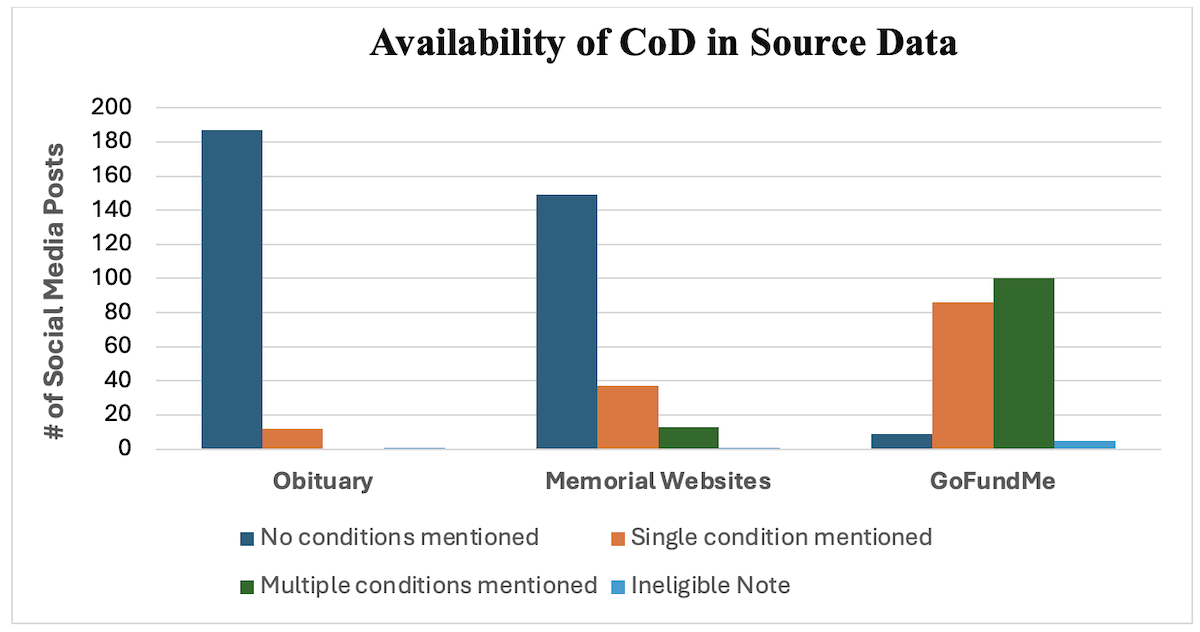

Supplement: Multimedia Appendix 2 [file jmir-v27-e71113-s002.png]
